# Age, Sex and Cerebral Microbleed Effects On White Matter Degradation After Traumatic Brain Injury

**DOI:** 10.1093/geroni/igaa057.3272

**Published:** 2020-12-16

**Authors:** Andrei Irimia, Ammar Dharani, Van Ngo, David Robles, Kenneth Rostowsky

**Affiliations:** University of Southern California, Los Angeles, California, United States

## Abstract

Mild traumatic brain injury (mTBI) affects white matter (WM) integrity and accelerates neurodegeneration. This study assesses the effects of age, sex, and cerebral microbleed (CMB) load as predictors of WM integrity in 70 subjects aged 18-77 imaged acutely and ~6 months after mTBI using diffusion tensor imaging (DTI). Two-tensor unscented Kalman tractography was used to segment and cluster 73 WM structures and to map changes in their mean fractional anisotropy (FA), a surrogate measure of WM integrity. Dimensionality reduction of mean FA feature vectors was implemented using principal component (PC) analysis, and two prominent PCs were used as responses in a multivariate analysis of covariance. Acutely and chronically, older age was significantly associated with lower FA (F2,65 = 8.7, p < .001, η2 = 0.2; F2,65 = 12.3, p < .001, η2 = 0.3, respectively), notably in the corpus callosum and in dorsolateral temporal structures, confirming older adults’ WM vulnerability to mTBI. Chronically, sex was associated with mean FA (F2,65 = 5.0, p = 0.01, η2 = 0.1), indicating males’ greater susceptibility to WM degradation. Acutely, a significant association was observed between CMB load and mean FA (F2,65 = 5.1, p = 0.009, η2 = 0.1), suggesting that CMBs reflect the acute severity of diffuse axonal injury. Together, these findings indicate that older age, male sex, and CMB load are risk factors for WM degeneration. Future research should examine how sex- and age-mediated WM degradation lead to cognitive decline and connectome degeneration after mTBI.

